# Serum sex hormone-binding globulin is a mediator of the association between intrahepatic lipid content and type 2 diabetes: the Maastricht Study

**DOI:** 10.1007/s00125-022-05790-7

**Published:** 2022-09-17

**Authors:** Pomme I. H. G. Simons, Olivier Valkenburg, Marjo P. H. van de Waarenburg, Marleen M. J. van Greevenbroek, M. Eline Kooi, Jacobus F. A. Jansen, Casper G. Schalkwijk, Coen D. A. Stehouwer, Martijn C. G. J. Brouwers

**Affiliations:** 1 Department of Internal Medicine, Division of Endocrinology and Metabolic Diseases, Maastricht University Medical Centre, Maastricht, the Netherlands; 2 Laboratory for Metabolism and Vascular Medicine, Maastricht University, Maastricht, the Netherlands; 3 CARIM School for Cardiovascular Diseases, Maastricht University, Maastricht, the Netherlands; 4 Department of Reproductive Medicine, Maastricht University Medical Centre, Maastricht, the Netherlands; 5 Department of Radiology and Nuclear Medicine, Maastricht University Medical Centre, Maastricht, the Netherlands; 6 School for Mental Health and Neuroscience, Maastricht University, Maastricht, the Netherlands; 7 Department of Electrical Engineering, University of Eindhoven, Eindhoven, the Netherlands; 8 Department of Internal Medicine, Division of General Internal Medicine, Maastricht University Medical Centre, Maastricht, the Netherlands

**Keywords:** Hepatokine, Mediation, Non-alcoholic fatty liver disease, Sex hormone-binding globulin, Type 2 diabetes

## Abstract

**Aims/hypothesis:**

Serum sex hormone-binding globulin (SHBG) has been proposed to act as a hepatokine that contributes to the extrahepatic complications observed in non-alcoholic fatty liver disease (NAFLD). However, it remains uncertain whether serum SHBG mediates the association between intrahepatic lipids (IHL) and type 2 diabetes. Therefore, we studied whether, and to what extent, serum SHBG mediates the association between IHL content and type 2 diabetes.

**Methods:**

We used cross-sectional data from the Maastricht Study (*n*=1554), a population-based cohort study with oversampling of individuals with type 2 diabetes. Type 2 diabetes status was assessed by oral glucose tolerance test, and IHL content was measured using 3T Dixon MRI. Mediation analyses were performed to assess the role of serum SHBG in mediating the association between IHL content and type 2 diabetes.

**Results:**

IHL content was significantly associated with type 2 diabetes in women and men (OR 1.08 [95% CI 1.04, 1.14] and OR 1.12 [95% CI 1.08, 1.17], respectively). Serum SHBG significantly mediated the association between IHL content and type 2 diabetes. The contribution of serum SHBG was higher in women (OR 1.04 [95% CI 1.02, 1.07]; proportion mediated 50.9% [95% CI 26.7, 81.3]) than in men (OR 1.02 [95% CI 1.01, 1.03]; proportion mediated 17.2% [95% CI 9.6, 27.6]). Repeat analyses with proxies of type 2 diabetes and adjustment for covariates did not substantially affect the results.

**Conclusions/interpretation:**

In this large-scale population-based cohort study, serum SHBG was found to be a mediator of the association between IHL content and type 2 diabetes. These findings extend our understanding of the potential mechanisms by which NAFLD is a risk factor for type 2 diabetes, and further elaborate on the role of SHBG as a hepatokine.

**Graphical abstract:**

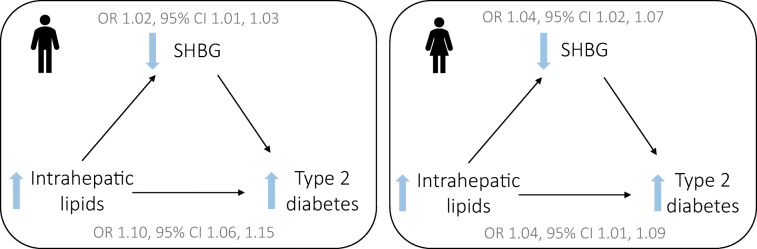

**Supplementary Information:**

The online version contains peer-reviewed but unedited supplementary material available at 10.1007/s00125-022-05790-7

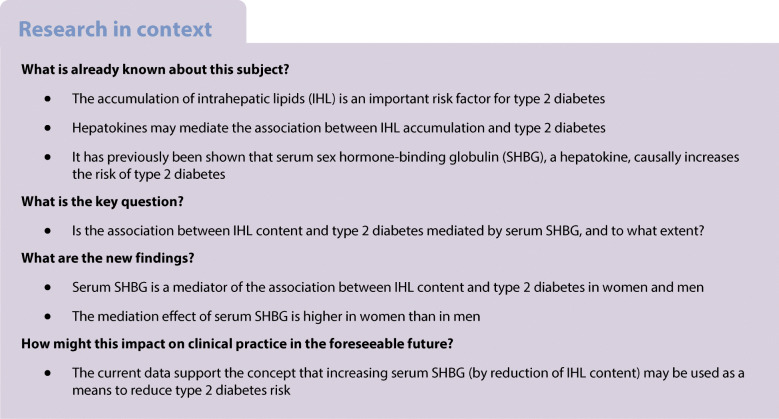



## Introduction

Non-alcoholic fatty liver disease (NAFLD) is a serious health concern that affects approximately 25% of the global population [1]. It encompasses a spectrum of histological abnormalities that result from excess storage of intrahepatic lipids (IHL) [2]. NAFLD is a precursor for several hepatic complications including liver failure and hepatocellular carcinoma, and is also a risk factor for the development of various extrahepatic complications, such as type 2 diabetes and cardiovascular disease [3, 4].

It has been proposed that hepatokines (liver-derived proteins that have systemic metabolic effects) may, in part, mediate the association between IHL accumulation and extrahepatic complications [5, 6]. Serum sex hormone-binding globulin (SHBG) has emerged as a hepatokine [7, 8]. SHBG is a glycoprotein that is synthesised in the liver under the regulation of several transcription factors including hepatocyte nuclear factor 4α, constitutive androstane receptor, peroxisome proliferator-activated receptor γ and chicken ovalbumin upstream promotor transcription factor [9–11]. In turn, these transcription factors are affected by several metabolic, hormonal, inflammatory and nutritional factors. Experimental studies have shown that de novo lipogenesis, the principal pathway resulting in accumulation of IHL [12], downregulates hepatocyte nuclear factor 4α, resulting in reduced SHBG expression [13]. We have previously extended these experimental data by showing that de novo lipogenesis, assessed using stable isotopes [12], is inversely associated with serum SHBG levels in humans [14].

Of interest, Mendelian randomisation studies have shown that genetically predicted low serum SHBG levels are associated with a higher risk of type 2 diabetes [15, 16]. This effect appears to be attributable to SHBG itself, i.e. independent of the effects of SHBG on free testosterone levels [17].

However, it remains to be elucidated to what extent serum SHBG mediates the association between IHL accumulation and type 2 diabetes. Therefore, the aim of the present study was to assess whether, and to what extent, serum SHBG has a mediating role in the association between IHL content and type 2 diabetes in a population-based cohort.

## Methods

### Study population

The Maastricht Study is a population-based cohort study with oversampling of individuals with type 2 diabetes. The study design and rationale have been extensively described previously [18]. In brief, the Maastricht Study focuses on the aetiology, pathophysiology, complications and comorbidities of type 2 diabetes, and involves extensive phenotyping of all participants. All individuals between 40 and 75 years of age living in the southern part of the Netherlands were eligible for participation. Participants were recruited through mass media campaigns and via mailings from the municipal registries and the regional Diabetes Patient Registry.

**Fig. 1:**
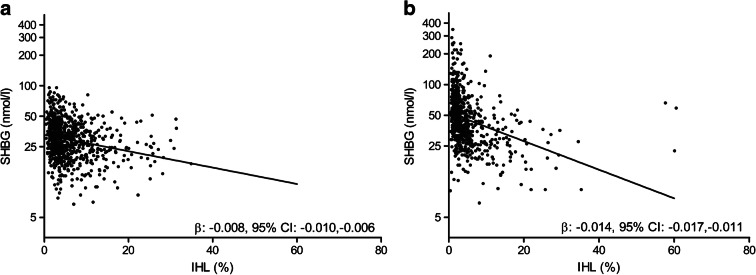
Association between IHL content and serum SHBG (logarithmic scale) in men (**a**) and women (**b**). The black line represents the line of best fit

The present study includes cross-sectional data from 3340 participants in whom serum SHBG levels were measured and who completed baseline measurements between November 2010 and December 2017. Quantification of IHL content was implemented from December 2013 onwards. Participants were excluded from the current analyses if they were diagnosed with other types of diabetes (*n*=41) or had missing data for IHL content (*n*=1161) or covariates (*n*=584). This resulted in a study population of 1554 participants (see electronic supplementary material [ESM] Fig. 1), of whom 369 had type 2 diabetes.

The Maastricht Study has been approved by the institutional medical ethical committee (NL31329.068.10) and the Minister of Health, Welfare and Sports of the Netherlands (permit 131088-105234-PG). All participants gave written informed consent prior to participation.

### Outcome: type 2 diabetes

All participants underwent a standardised 2 h 75 g oral glucose tolerance test after an overnight fast, except for individuals using insulin and/or individuals with a fasting capillary glucose ≥ 11.1 mmol/l. For these individuals, information about fasting glucose levels, use of glucose-lowering medication, and history of other types of diabetes was used to assess type 2 diabetes status [18]. Diabetes was defined according to the WHO 2006 diagnostic criteria as a fasting plasma glucose ≥ 7.0 mmol/l and/or a 2 h plasma glucose ≥ 11.1 mmol/l [19].

### Exposure: IHL content

IHL content was quantified by Dixon MR imaging using a 3.0 Tesla MRI system (MAGNETOM Prismafit, Siemens, Germany) with body matrix and supine radiofrequency coils. After a scout scan, transverse two-dimensional T2-weighted true fast imaging with steady-state free precession (T2w TRUFI) images of the liver were obtained using the following variables: voxel size: 1.2 × 1.2 × 5.0 mm^3^, repetition time 422 ms, echo time 1.65 ms, flip angle 60°, number of signal averages 1, parallel imaging (GRAPPA) factor 2. Next, transverse two-dimensional turbo spin echo Dixon MR images were obtained of the liver during a breath-hold using the following variables: voxel size: 2.0 × 2.0 × 6.0 mm^3^, number of slices 4, repetition time 500 ms, echo time 31 ms, turbo factor 5, number of signal averages 1, parallel imaging (GRAPPA) factor 3 [20]. Three regions of interest (ROIs) in the liver were drawn on the T2w TRUFI images by trained observers, taking care to position the ROIs in artifact-free regions and to avoid positioning the ROIs on visible structures, such as vessels and bile ducts. Subsequently, these ROIs were copied to the water and fat Dixon MR images to calculate the IHL percentage, expressed as the ratio CH_2_/H_2_O × 100%. Hepatic steatosis has been defined as an IHL content >5.56% when expressed as CH_2_/(H_2_O + CH_2_) [21], which corresponds to a cut-off of 5.89% (0.0556/(1–0.0556)) when IHL is expressed as CH_2_/H_2_O, as was done in this study.

The Dixon MRI method was validated in 36 participants with a broad range of IHL content, and calibrated against the results obtained using 3T proton magnetic resonance spectroscopy (^1^H-MRS), i.e. the gold standard for non-invasively quantifying IHL [22]. After calibration, the intraclass correlation coefficient between Dixon MRI and ^1^H-MRS was 0.989 (95% CI 0.979, 0.994).

### Mediator: serum SHBG levels

Serum SHBG levels were measured using a human SHBG DuoSet solid-phase sandwich ELISA (R&D Systems, USA) according to the manufacturer’s instructions. The intra- and interassay coefficients of variation for serum SHBG were 2.8% and 5.1%, respectively. The DuoSet ELISA was validated against a chemiluminescent immunometric assay (Immulite XPi, Siemens, Germany) in eight samples. The intraclass correlation coefficient was 0.974 (95% CI 0.862, 0.995).

### Measurement of covariates

All participants completed questionnaires regarding age, sex, educational level (low, medium or high), smoking status (never, former or current smoker), use of alcohol (g/day) and menopausal status (postmenopausal status was defined as a most recent menstrual period more than 12 months prior to the time of assessment) [18]. Use of medication was assessed through medication interviews. Anthropometric measurements including weight, height, waist circumference and office systolic and diastolic blood pressure were measured during physical examination. BMI was calculated as weight (kg) divided by height (m) squared [18]. Daily total physical activity levels were measured during 8 consecutive days using activPAL3 physical activity monitors (PAL Technologies, UK) and expressed as minutes of stepping activity per day [23]. Fasting levels of glucose, insulin, HbA_1c_ and lipid profile (total cholesterol, HDL-cholesterol, LDL-cholesterol and triglycerides) were measured in venous blood samples [18]. Insulin sensitivity was estimated using the Matsuda insulin sensitivity index [24]. Adherence to the Dutch dietary guidelines was assessed based on the Dutch Healthy Diet (DHD) index consisting of 15 components and based on food frequency questionnaires [25]. In the Maastricht Study, the DHD index consists of 14 components (DHD-14). The coffee component was not included as it is based on the type of coffee consumed, which the food frequency questionnaires were unable to distinguish between [26]. Furthermore, as we included alcohol consumption as a separate covariate in the regression models (see below), the DHD index in the present study was reported as the DHD-13 (DHD-14 minus the alcohol component).

### Statistical analyses

Continuous data are presented as mean ± SD, or as median (IQR) in the case of a non-normal distribution. Categorical data are presented as percentages. Non-normally distributed variables were log_10_-transformed prior to further analyses.

Univariate regression analyses were performed to study the associations between (1) IHL content and serum SHBG; (2) serum SHBG and type 2 diabetes (crude and adjusted for IHL content); and (3) IHL content and type 2 diabetes. All associations were explored for an interaction with sex. Regression coefficients are presented as unstandardised β coefficients.

Mediation analyses were then performed to investigate whether the association between IHL content and type 2 diabetes status was mediated by serum SHBG, as illustrated in the directed acyclic graph in ESM Fig. 2. We also tested for an effect of an interaction between the exposure and the mediator on the outcome. The mediation analyses were adjusted for the following confounders: model 1 was adjusted for age; model 2 was additionally adjusted for (proxies of) lifestyle: BMI, alcohol intake, DHD-13, level of education and total physical activity; model 3 was additionally adjusted for menopausal status and use of oestrogen-containing medication in women. Furthermore, given the oversampling of participants with type 2 diabetes, analyses were repeated after taking the higher prevalence of type 2 diabetes into consideration using case–control mediation analyses [27]. The analyses were then repeated using a binary exposure, i.e. hepatic steatosis yes/no. Lastly, to further test the robustness of our findings, the analyses were repeated with adjustment for waist circumference instead of BMI (model 2), and analyses were repeated with proxies of type 2 diabetes as the outcome variable, i.e. HbA_1c_ and the Matsuda index.

For all mediation analyses, the regression-based approach [28, 29] was used to estimate the natural direct and natural indirect effects. The 95% CIs were estimated using non-parametric bootstrapping with the percentile method. The proportion mediated (%) was estimated as OR^Direct^ × (OR^Indirect^ – 1)/(OR^Direct^ × OR^Indirect^ – 1) × 100 in the case of a binary outcome [30], or as β^Indirect^/β^Total^ × 100 in the case of a continuous outcome [31]. All results were considered statistically significant at a *p* value<0.05, except for interaction terms where a less stringent *p* value threshold was considered statistically significant (*p*<0.10).

Statistical analyses were performed using SPSS version 27.0 for Windows (IBM, USA) and R statistical software version 4.0.1 (R Foundation for Statistical Computing, Austria) with the CMAverse package [32].

## Results

### Study population

Table 1 shows the characteristics of the overall study population and the population stratified according to type 2 diabetes status. The overall population had a mean age of 60 ± 8 years and 47.9% were female, of whom the majority (79.2%) were postmenopausal. Only a small number of women (4.0%) used oestrogen-containing medication. The overall population had a median IHL content of 3.5% (IQR 2.1–6.5) and a median serum SHBG level of 35.5 nmol/l (IQR 25.3–49.8). Although Table 1 data were not tested for statistical significance, participants with type 2 diabetes were more often male, tended to be older, and generally had a poorer metabolic profile (i.e. higher BMI, waist circumference, systolic blood pressure and serum triglycerides, and lower HDL-cholesterol and Matsuda index). Furthermore, participants with type 2 diabetes had a higher median IHL content and lower serum SHBG levels.

**Table 1:** Characteristics of the overall study population and the population stratified according to type 2 diabetes status

Variable	Overall (*n*=1554)	Individuals without type 2 diabetes (*n*=1185)	Individuals with type 2 diabetes (*n*=369)
Age, years	60 ± 8	59 ± 8	62 ± 8
Sex, % women	47.9	53.8	29.0
Postmenopausal, % of women	79.2	78.3	84.1
Use of oestrogen-containing medication, % of women	4.0	3.8	5.6
Education level low/medium/high, %	30.3/28.8/40.9	27.3/28.4/44.2	39.8/30.1/30.1
Smoking, never/former/current, %	38.0/50.2/11.8	39.0/49.3/11.7	34.8/53.0/12.2
DHD index (DHD-13)^a^	77.2 ± 13.9	78.0 ± 13.9	74.8 ± 13.7
Alcohol, g/day	9.0 (2.0–19.0)	9.8 (2.7–19.5)	5.8 (0.5–16.0)
Physical activity, min/day	120.8 (93.6–148.7)	125.5 (101.1–152.5)	100.9 (74.6–135.8)
BMI, kg/m^2^	26.6 ± 4.1	25.8 ± 3.7	29.0 ± 4.3
Waist circumference, cm	94.3 ± 12.5	91.3 ± 11.0	103.8 ± 12.5
Office systolic blood pressure, mmHg	134 ± 17	132 ± 17	140 ± 16
Office diastolic blood pressure, mmHg	76 ± 10	76 ± 10	77 ± 9
Total cholesterol, mmol/l	5.3 ± 1.2	5.6 ± 1.1	4.5 ± 1.0
HDL-cholesterol, mmol/l	1.6 ± 0.5	1.7 ± 0.5	1.3 ± 0.4
LDL-cholesterol, mmol/l	3.1 ± 1.0	3.3 ± 1.0	2.4 ± 0.9
Triglycerides, mmol/l	1.2 (0.9–1.7)	1.1 (0.8–1.5)	1.5 (1.1–2.1)
Use of lipid-modifying medication, %	32.3	19.7	72.9
HbA_1c_, %	5.6 (5.4–6.0)	5.4 (5.3–5.7)	6.7 (6.2–7.4)
HbA_1c_, mmol/mol	38.0 (35.0–42.0)	36.0 (34.0–39.0)	50.0 (44.5–57.0)
Fasting glucose, mmol/l	5.5 (5.0–6.3)	5.3 (4.9–5.7)	7.5 (6.8–8.6)
Fasting insulin, mmol/l	59.1 (41.8–87.8)	55.3 (40.0–77.0)	83.2 (51.3–126.0)
Matsuda index	3.6 (2.1–5.3)	4.1 (2.6–5.8)	2.0 (1.3–3.0)
Use of glucose-lowering medication, %	17.9	0.0	75.3
IHL content, %	3.5 (2.1–6.5)	2.9 (1.9–5.1)	5.2 (3.5–10.7)
Hepatic steatosis, % yes	29.9	22.0	55.0
Serum SHBG, nmol/l	35.5 (25.3–49.8)	38.7 (27.6–54.2)	26.4 (19.6–37.0)

Data are presented as mean ± SD or median (IQR) unless otherwise indicated

^a^Dutch Healthy Diet (DHD-14) index minus alcohol component (DHD-13)

### Univariate regression analyses

Univariate regression analyses were performed to study the association between (1) IHL content and serum SHBG levels (i.e. exposure–mediator); (2) IHL content and type 2 diabetes status (i.e. exposure–outcome); and (3) serum SHBG levels and type 2 diabetes status (i.e. mediator–outcome). As there was a statistically significant interaction effect of the dependent variable and sex on the outcome for all three associations (*p*=0.001, 0.040 and < 0.001, respectively), all analyses were subsequently stratified according to sex. The sex-stratified population characteristics are presented in ESM Table 1.

There was a statistically significant inverse association between IHL content and serum SHBG levels in men and women (β −0.008 [95% CI −0.010, −0.006] and β −0.014 [95% CI −0.017, −0.011], respectively) (Fig. 1). The strength of association was stronger in women, and this remained after exclusion of premenopausal women and women using oestrogen-containing medication (β −0.015 [95% CI −0.018, −0.012]). Furthermore, there was a statistically significant association between IHL content and type 2 diabetes status in men and women (OR 1.12 [95% CI 1.08, 1.17] and OR 1.08 [95% CI 1.04, 1.14], respectively). Of note, these associations represent the total effect estimates in the mediation analyses (i.e. exposure–outcome). Lastly, there was a statistically significant inverse association between serum SHBG and type 2 diabetes status in men and women (OR 0.96 [95% CI 0.95, 0.98] and OR 0.98 [95% CI 0.97, 0.99], respectively). Adjustment for IHL content did not affect these associations (OR 0.97 [95% CI 0.96, 0.98] and OR 0.98 [95% CI 0.97, 0.99], respectively). Furthermore, for all analyses, adjustment for age, BMI, alcohol intake, DHD-13, level of education, physical activity, and menopausal status and use of oestrogen-containing medication in women, did not substantially affect the results (*p*<0.025 for all analyses; data not shown).

### Mediation analyses

**Fig. 2:**
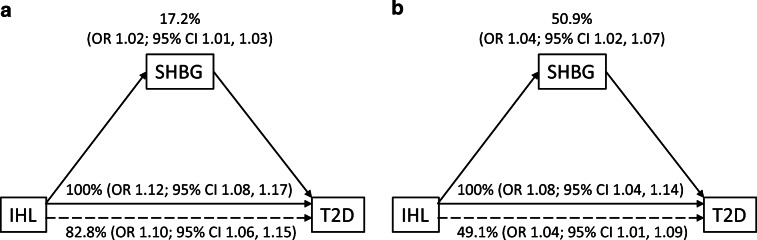
Crude association between IHL content and type 2 diabetes (T2D) mediated by serum SHBG in men (**a**) and women (**b**). Solid horizontal arrows represent the total effect, i.e. the association between IHL content and type 2 diabetes status. Dashed horizontal arrows indicate the direct effect, i.e. the association between IHL content and type 2 diabetes status that is not attributable to serum SHBG

We performed mediation analyses to assess whether, and to what extent, the relationship between IHL content and type 2 diabetes status was mediated by serum SHBG levels. There was no interaction effect of the exposure and the mediator on the outcome in both men and women. We found that the association between IHL content and type 2 diabetes was statistically significantly mediated by serum SHBG in men (OR 1.02 [95% CI 1.01, 1.03]) and women (OR 1.04 [95% CI 1.02, 1.07]) (Fig. 2). In men, serum SHBG was estimated to mediate 17.2% (95% CI 9.6, 27.6) of the association between IHL content and type 2 diabetes, while the proportion mediated was 50.9% (95% CI 26.7, 81.3) in women. The mediation effect of serum SHBG remained statistically significant after adjustment for age (model 1), BMI, alcohol intake, DHD-13, level of education and total physical activity (model 2), and menopausal status and use of oestrogen-containing medication in women (model 3) (Table 2).

**Table 2:** Mediation effect of serum SHBG on the association between IHL content and type 2 diabetes

Model	Men (*n*=810)		Women (*n*=744)	
	OR (95% CI)	Proportion mediated (95% CI)^a^	OR (95% CI)	Proportion mediated (95% CI)^a^
Crude				
Total effect^b^	1.12 (1.08, 1.17)		1.08 (1.04, 1.14)	
Direct effect^b^	1.10 (1.06, 1.15)		1.04 (1.01, 1.09)	
Indirect effect^b^	1.02 (1.01, 1.03)	17.2 (9.6, 27.6)	1.04 (1.02, 1.07)	50.9 (26.7, 81.3)
Model 1				
Total effect	1.13 (1.09, 1.17)		1.08 (1.05, 1.13)	
Direct effect	1.10 (1.06, 1.14)		1.04 (1.01, 1.09)	
Indirect effect	1.03 (1.02, 1.04)	24.6 (15.6, 36.0)	1.04 (1.02, 1.07)	48.5 (24.0, 80.4)
Model 2				
Total effect	1.08 (1.05, 1.13)		1.04 (1.00, 1.09)	
Direct effect	1.07 (1.03, 1.11)		1.02 (0.98, 1.07)	
Indirect effect	1.01 (1.01, 1.02)	17.7 (8.3, 32.8)	1.02 (1.00, 1.04)	42.6 (2.5, 254.1)
Model 3				
Total effect			1.04 (1.00, 1.10)	
Direct effect			1.02 (0.98, 1.07)	
Indirect effect			1.02 (1.01, 1.04)	55.9 (−72.38, 337.2)

Model 1 was adjusted for age; model 2 was additionally adjusted for BMI, alcohol intake, DHD-13, level of education and total physical activity; model 3 was additionally adjusted for menopausal status and use of oestrogen-containing medication in women

^a^The proportion mediated (%) is calculated as OR^Direct^ × (OR^Indirect^ − 1)/(OR^Direct^ × OR^Indirect^ − 1) × 100

^b^Total effect represents association between IHL content and type 2 diabetes status; direct effect represents association between IHL and type 2 diabetes status not attributable to serum SHBG; indirect effect represents association between IHL and type 2 diabetes attributable to serum SHBG (mediation)

### Additional analyses

The mediation analyses were repeated after accounting for the oversampling of participants with type 2 diabetes in the Maastricht Study; this did not substantially affect the results (ESM Table 2). In addition, the analyses were repeated using a binary exposure, i.e. hepatic steatosis (yes/no). This did not materially affect the results, although statistical significance was lost in model 2 for men (*p*=0.16; ESM Table 3). In addition, the mediation analyses were repeated after adjustment for waist circumference instead of BMI (model 2); this did not materially change the results (ESM Table 4). Finally, mediation analyses were repeated using proxies for type 2 diabetes (i.e. HbA_1c_ and the Matsuda index) as the dependent variable. In both men and women, serum SHBG remained a statistically significant mediator in the association between IHL content and both HbA_1c_ and the Matsuda index in the crude and fully adjusted models (ESM Tables 5 and 6).

## Discussion

In the present study, serum SHBG partially mediated the association between the IHL content and type 2 diabetes status. The mediating role of serum SHBG in the association between IHL content and type 2 diabetes was more substantial in women than in men. Similar results were found when the analyses were repeated using proxies of type 2 diabetes (i.e. HbA_1c_ and the Matsuda index) and when adjusted for confounders.

The importance of hepatokines in the pathogenesis of extrahepatic disease, in particular type 2 diabetes, is increasingly recognised [5, 6, 33]. Nevertheless, this is the first study that has assessed the mediation effect of serum SHBG in the association between IHL content and type 2 diabetes. The current findings corroborate the hypothesis that SHBG may have a role not only as carrier protein for testosterone and a biomarker of metabolic disease, but also as a hepatokine affecting type 2 diabetes [16, 17, 33]. Experimental studies have shown that de novo lipogenesis, which is one of the primary pathways contributing to the accumulation of IHL [12], downregulates hepatocyte nuclear factor 4α and subsequently serum SHBG levels [13], a finding that we recently extrapolated to humans [14, 34]. Mendelian randomisation studies have shown that genetically predicted low serum SHBG levels are causally associated with an increased risk of type 2 diabetes, independent of the effects of SHBG on free testosterone levels [15–17, 35]. However, the exact biological mechanism by which serum SHBG influences type 2 diabetes is poorly understood, and experimental studies into the mechanism of action are scarce and sometimes contradictory [36–38]. These studies are complicated by the fact that wild-type rodents do not express hepatic SHBG, which limits the extrapolation to humans. Therefore, further research to unravel the exact mechanisms through which SHBG exerts its effects is needed.

It is likely that there are several pathways that mediate the association between IHL content and type 2 diabetes, of which serum SHBG is merely one. Insulin resistance and excess (hepatic) glucose production are other well-known mediators [39]. The accumulation of IHL contributes to an excess of circulating fatty acid metabolites in peripheral tissues, which are involved in the pathogenesis of insulin resistance [6, 39]. In addition, the carbohydrate regulatory element binding protein, which is one of the principal transcription factors that regulate de novo lipogenesis [40], activates glucose-6-phosphatase and thereby contributes to increased hepatic glucose production [41]. It is likely that these pathways are largely responsible for the remaining direct effect of IHL content on type 2 diabetes that was observed in this study.

We observed a relatively high estimated proportion of mediation by serum SHBG in the association between IHL content and type 2 diabetes. This may be an indication of the biological relevance of serum SHBG in the pathogenesis of type 2 diabetes. Indeed, the indirect effects of our mediation analyses suggest that a single percentage point increase in IHL content is associated with 8–12% higher odds of type 2 diabetes overall and 2–4% higher odds of type 2 diabetes when mediated via serum SHBG. Given that the median IHL content in this study population was 3.5% (IQR 2.1–6.5) (Table 1), the current findings are likely to be clinically relevant. Nevertheless, in some analyses, there was a considerable uncertainty in the estimated proportion mediated, particularly when the direct effect (i.e. the effect of IHL content on type 2 diabetes that was not attributable to serum SHBG) was not statistically significant. This may indicate methodological limitations of the estimated proportion mediated, and the results should therefore be interpreted with care [42]. Furthermore, the high estimated proportion mediated may partially be the result of potential bi-directionality of the associations. Although it is assumed that there is a causal association between IHL content, serum SHBG and type 2 diabetes (an assumption that is supported by experimental and genetic studies [13, 15, 16]) we cannot exclude the possibility that the associations are in fact bi-directional. Indeed, experimental studies have found that transgenic *SHBG* mice show reduced IHL content and improved glucose homeostasis [36, 37, 43], although these findings have not yet been extrapolated to humans. In addition, hyperinsulinaemia and hyperglycaemia, which are characteristic of type 2 diabetes [44], stimulate de novo lipogenesis and consequently IHL accumulation [45]. As a result of the bi-directionality of these associations, the observed estimates should be regarded as the maximum mediation effects.

There was a noticeable difference in the mediation effect of serum SHBG between men and women, with a higher contribution observed in women. Of interest, previous observational studies have reported similar sexually dimorphic associations between IHL content and serum SHBG [7], in line with the results of the univariate regression analyses in this study [8, 15]. Moreover, genetic studies have reported that variants in the glucokinase regulator gene *GCKR*, which are associated with higher rates of de novo lipogenesis and IHL content [46], have a stronger, inverse association with serum SHBG in women than in men [47]. However, the biological mechanisms that account for these sex differences remain poorly understood and deserve further investigation.

This study has several strengths. By using data from the Maastricht Study, we were able to obtain a large cohort of individuals with oversampling of participants with type 2 diabetes. The extensive phenotyping allowed for adjustment for many well-defined confounders, such as use of oestrogen-containing medication and physical activity, as assessed using an accelerometer. Furthermore, IHL content was quantified using state-of-the-art methodology (i.e. Dixon MRI). This study also had several limitations. First, as a result of the cross-sectional nature of the data, we cannot draw conclusions on causality. Although experimental and genetic studies support the assumptions of causality in this study, we cannot exclude the possibility that, as mentioned above, the associations are bi-directional. Furthermore, although we adjusted for the most important confounders, we cannot exclude the possibility that there may be residual confounding. For instance, other unmeasured confounders such as endogenous oestrogens, thyroid hormone or adipokines, which all affect IHL content, serum SHBG levels and type 2 diabetes, may confound the current mediation analyses. Adjustment for these unmeasured confounders may reduce the strength of the observations. Furthermore, we cannot exclude the possibility that other hepatokines, which are also associated with IHL content [33], also mediate the association between IHL content and type 2 diabetes status [48]; this deserves further investigation. Lastly, the participants in the current study were primarily of European descent, aged between 40 and 75 years, which resulted in a relatively low number of premenopausal women. Caution should therefore be exercised when extrapolating to other groups.

In conclusion, in a large-scale population-based cohort study, we show that serum SHBG is a mediator in the association between IHL content and type 2 diabetes. The mediation effect was larger in women. These findings extend our knowledge on the potential mechanisms that link NAFLD with type 2 diabetes, and emphasise the importance of serum SHBG as a hepatokine. Furthermore, the current data support the concept that increasing serum SHBG (by reduction of IHL content) may be used as a means to reduce type 2 diabetes risk.

## Supplementary information

ESM (PDF 582 KB)

## Data Availability

Data are available upon reasonable request and with permission from the Maastricht Study management team.

## Abbreviations

DHD: Dutch Healthy Diet
IHL: Intrahepatic lipid
NAFLD: Non-alcoholic fatty liver disease
ROI: Region of interest
SHBG: Sex hormone-binding globulin
