# Supplementary material for: Serum sex hormone-binding globulin is a mediator of the association between intrahepatic lipid content and type 2 diabetes: the Maastricht Study
Source: Diabetologia. 2022 Sep 17;66(1):213–22. doi: 10.1007/s00125-022-05790-7 (PMC9729158; doi:10.1007/s00125-022-05790-7)
Supplement: Supplementary file 1 — (PDF 582 KB) [file 125_2022_5790_MOESM1_ESM.pdf]

Serum sex hormone-binding globulin is a mediator of the association between intrahepatic lipid content and type 2 diabetes: The Maastricht Study

Electronic Supplementary Materials

ESM Table 1. Characteristics of the study population stratified according to sex

	Men			Women		
	**Overall (n = 810)**	**Individuals without type 2 diabetes (n = 548)**	**Individuals with type 2 diabetes (n = 262)**	**Overall (n = 744)**	**Individuals without type 2 diabetes (n = 637)**	**Individuals with type 2 diabetes (n = 107)**
Age, years	61 ± 8	60 ± 8	63 ± 8	58 ± 8	58 ± 8	61 ± 8
Postmenopausal, % of women				79.2	78.3	84.1
Use of oestrogen-containing medication, % of women				4.0	3.8	5.6
Education level low/medium/high, %	25.8 / 29.6 / 44.6	21.2 / 29.4 / 49.5	35.5 / 30.2 / 34.4	35.2 / 28.0 / 36.8	32.7 / 27.6 / 39.7	50.5 / 29.9 / 19.6
Smoking, never/former/current, %	36.7 / 52.0 / 11.2	38.1 / 50.7 / 11.1	33.7 / 54.8 / 11.5	39.4 / 48.1 / 12.5	39.7 / 48.0 / 12.2	42.8 / 48.6 / 14.0
Dutch healthy diet index (DHD-13)*	73.9 ± 13.9	74.3 ± 14.0	74.1 ± 13.6	80.9 ± 13.0	81.2 ± 13.0	78.9 ± 12.9
Alcohol, g/day	12.0 (3.9-23.5)	14.3 (5.6-24.7)	7.6 (1.5-20.7)	6.0 (0.8-14.2)	7.1 (1.3-14.7)	5.2 (0.1-8.8)
Physical activity, min/day	119.4 (88.3-149.4)	126.8 (97.1-154.9)	99.0 (72.0-135.3)	122.7 (100.3-148.3)	127.8 (103.4-150.1)	105.0 (80.8-137.0)
BMI, kg/m^2	27.2 ± 3.7	26.3 ± 3.1	28.7 ± 4.1	25.9 ± 4.4	25.3 ± 4.1	28.9 ± 4.9
Waist circumference, cm	99.8 ± 11.0	96.8 ± 9.3	106.1 ± 11.5	88.3 ± 11.4	86.7 ± 10.2	98.2 ± 13.0
Office systolic blood pressure, mmHg	139 ± 16	137 ± 17	142 ± 16	128 ± 16	127 ± 16	135 ± 17
Office diastolic blood pressure, mmHg	79 ± 10	79 ± 10	78 ± 10	73 ± 9	73 ± 9	74 ± 8
Total cholesterol, mmol/L	5.0 ± 1.1	5.4 ± 1.0	4.3 ± 1.0	5.6 ± 1.1	5.8 ± 1.1	4.7 ± 1.1
HDL cholesterol, mmol/L	1.4 ± 0.4	1.5 ± 0.4	1.2 ± 0.3	1.8 ± 0.5	1.8 ± 0.5	1.5 ± 0.4

LDL cholesterol, mmol/L	3.0 ± 1.0	3.3 ± 0.9	2.3 ± 0.9	3.2 ± 1.0	3.4 ± 1.0	2.5 ± 0.9
Triglycerides, mmol/L	1.3 (0.9-1.8)	1.2 (0.9-1.6)	2.2 (1.7-2.8)	1.1 (0.8-1.6)	1.1 (0.8-1.5)	1.5 (1.1-2.2)
Use of lipid-modifying medication, %	40.6	24.3	74.8	23.3	15.7	68.2
HbA1c, %	5.6 (5.4-6.3)	5.4 (5.3-5.7)	6.7 (6.3-7.3)	5.5 (5.3-5.8)	5.4 (5.3-5.7)	6.6 (6.0-7.4)
HbA1c, mmol/mol	38 (35-45)	36 (34-39)	50 (45-56)	37 (34-40)	36 (34-39)	49 (42-57)
Fasting glucose, mmol/L	5.8 (5.2-6.9)	5.4 (5.1-5.9)	7.7 (6.9-8.9)	5.2 (4.9-5.7)	5.1 (4.9-5.5)	7.3 (6.5-8.0)
Fasting insulin, pmol/L	64.3 (45.1-98.3)	60.8 (44.1-86.8)	83.3 (47.4-129.0)	53.3 (38.3-75.9)	51.0 (37.2-69.8)	82.8 (54.4-118.1)
Matsuda index	3.1 (1.9-4.7)	3.5 (2.3-5.1)	2.0 (1.2-3.0)	4.3 (2.6-6.0)	4.5 (3.1-6.2)	2.0 (1.4-2.7)
Use of glucose-lowering medication, %	24.8	0.0	76.7	10.3	0	72.0
Intrahepatic lipid content, %	4.0 (2.5-7.5)	3.5 (2.3-6.0)	6.5 (3.6-10.9)	2.7 (1.7-5.2)	2.5 (1.6-4.5)	5.5 (3.3-10.2)
Serum SHBG, nmol/L	29.6 (22.4-39.7)	31.8 (24.1-41.8)	25.4 (19.1-32.5)	45.0 (31.8-64.9)	47.3 (35.0-66.0)	31.9 (21.5-47.5)

* Dutch Healthy Diet (DHD-14) index minus alcohol component (= DHD-13).

Data are presented as mean ± standard deviation or median (interquartile range), unless otherwise indicated.

Abbreviations: BMI: body mass index; HDL: high density lipoprotein; LDL: low density lipoprotein; HbA1c: haemoglobin A1c; SHBG: sex hormone-binding globulin

ESM Table 2. Mediation effect of serum SHBG on the association between intrahepatic lipid content and type 2 diabetes status after accounting for oversampling of individuals with type 2 diabetes

	**Men (n = 810)**		**Women (n = 744)**	
	**OR (95% CI)**	**% mediated (95% CI)***	**OR (95% CI)**	**% mediated (95% CI)***
**Crude**				
Total effect†	1.12 (1.08;1.16)		1.07 (1.04;1.14)	
Direct effect†	1.10 (1.07;1.15)		1.04 (1.01;1.09)	
Indirect effect†	1.01 (1.01;1.02)	12.3 (5.5;21.3)	1.03 (1.02;1.06)	47.8 (25.5;78.8)
**Model 1**				
Total effect	1.12 (1.08;1.16)		1.07 (1.04;1.13)	
Direct effect	1.10 (1.06;1.14)		1.04 (1.01;1.09)	
Indirect effect	1.02 (1.01;1.03)	18.5 (9.7;29.0)	1.03 (1.02;1.06)	45.6 (23.0;79.0)
**Model 2**				
Total effect	1.08 (1.04;1.13)		1.04 (1.00;1.09)	
Direct effect	1.07 (1.03;1.11)		1.02 (0.98;1.07)	
Indirect effect	1.01 (1.00;1.02)	12.0 (0.5;26.6)	1.01 (1.00;1.09)	39.7 (-15.3;268.5)
**Model 3**				
Total effect			1.04 (1.00;1.09)	
Direct effect			1.02 (0.98;1.07)	
Indirect effect			1.02 (1.01;1.04)	53.2 (-142.2;332.5)

* % mediated is calculated as $OR^{Direct}(OR^{Indirect} - 1)/(OR^{Direct} * OR^{Indirect} - 1) * 100$

† Total effect represents association between intrahepatic lipid (IHL) content and type 2 diabetes; direct effect represents association between IHL and type 2 diabetes status not attributable to serum SHBG; indirect effect represents association between IHL and type 2 diabetes attributable to serum SHBG (= mediation).

Model 1: adjusted for age

Model 2: additionally adjusted for BMI, alcohol intake, Dutch Healthy Diet index (DHD-13), level of education, and total physical activity

Model 3: additionally adjusted for menopausal status and use of oestrogen-containing medication

ESM Table 3. Mediation effect of serum SHBG on the association between hepatic steatosis (yes/no) and type 2 diabetes status

	**Men (n = 810)**		**Women (n = 744)**	
	**OR (95% CI)**	**% mediated (95% CI)***	**OR (95% CI)**	**% mediated (95% CI)***
**Crude**				
Total effect†	3.62 (2.66;4.99)		4.41 (2.86;6.83)	
Direct effect†	3.22 (2.35;4.45)		2.29 (1.24;4.02)	
Indirect effect†	1.12 (1.03;1.23)	15.0 (4.3;25.9)	1.28 (0.88;1.86)	28.2 (-17.6;65.8)
**Model 1**				
Total effect	3.59 (2.61;5.01)		4.21 (2.71;6.57)	
Direct effect	2.98 (2.16;4.13)		2.65 (1.52;4.48)	
Indirect effect	1.21 (1.08;1.36)	23.6 (10.3;36.7)	1.59 (1.22;2.17)	48.7 (22.9;74.5)
**Model 2**				
Total effect	2.53 (1.79;3.66)		2.52 (1.52;4.23)	
Direct effect	2.35 (1.66;3.99)		2.01 (1.12;3.55)	
Indirect effect	1.08 (0.97;1.20)	11.7 (-5.31;27.4)	1.25 (1.04;1.59)	33.2 (5.2;80.1)
**Model 3**				
Total effect			2.56 (1.54;4.37)	
Direct effect			1.85 (1.03;3.27)	
Indirect effect			1.38 (1.15;1.75)	45.5 (20.0;94.3)

* % mediated is calculated as $OR^{Direct}(OR^{Indirect} - 1)/(OR^{Direct} * OR^{Indirect} - 1) * 100$

† Total effect represents association between hepatic steatosis (yes/no) and type 2 diabetes; direct effect represents association between hepatic steatosis and type 2 diabetes status not attributable to serum SHBG; indirect effect represents association between hepatic steatosis and type 2 diabetes attributable to serum SHBG (= mediation).

Model 1: adjusted for age

Model 2: additionally adjusted for BMI, alcohol intake, Dutch Healthy Diet index (DHD-13), level of education, and total physical activity

Model 3: additionally adjusted for menopausal status and use of oestrogen-containing medication

ESM Table 4. Mediation effect of serum SHBG on the association between intrahepatic lipid content and type 2 diabetes status with adjustment for waist circumference in model 2.

	**Men (n = 810)**		**Women (n = 744)**	
	**OR (95% CI)**	**% mediated (95% CI)***	**OR (95% CI)**	**% mediated (95% CI)***
**Crude**				
Total effect†	1.12 (1.09;1.17)		1.08 (1.04;1.14)	
Direct effect†	1.10 (1.07;1.15)		1.04 (1.01;1.09)	
Indirect effect†	1.02 (1.01;1.03)	17.2 (9.6;27.6)	1.04 (1.02;1.07)	50.9 (26.7;81.3)
**Model 1**				
Total effect	1.13 (1.09;1.18)		1.08 (1.05;1.13)	
Direct effect	1.10 (1.06;1.14)		1.04 (1.01;1.09)	
Indirect effect	1.03 (1.02;1.04)	24.6 (15.6;36.0)	1.04 (1.02;1.07)	48.5 (24.0;80.4)
**Model 2**				
Total effect	1.08 (1.04;1.12)		1.02 (0.98;1.07)	
Direct effect	1.06 (1.03;1.11)		1.01 (0.97;1.06)	
Indirect effect	1.01 (1.01;1.02)	18.0 (7.8;35.9)	1.01 (1.00;1.03)	49.5 (-45.3;481.0)
**Model 3**				
Total effect			1.02 (0.98;1.07)	
Direct effect			1.00 (0.96;1.05)	
Indirect effect			1.02 (1.00;1.04)	77.2 (-616.6;721.9)

* % mediated is calculated as $OR^{Direct}(OR^{Indirect} - 1)/(OR^{Direct} * OR^{Indirect} - 1) * 100$

† Total effect represents association between intrahepatic lipid (IHL) content and type 2 diabetes; direct effect represents association between IHL and type 2 diabetes status not attributable to serum SHBG; indirect effect represents association between IHL and type 2 diabetes attributable to serum SHBG (= mediation).

Model 1: adjusted for age

Model 2: additionally adjusted for waist circumference, alcohol intake, Dutch Healthy Diet index (DHD-13), level of education, and total physical activity
Model 3: additionally adjusted for menopausal status and use of oestrogen-containing medication

ESM Table 5. Mediation effect of serum SHBG on the association between intrahepatic lipids and (log) HbA1c

	**Men (n = 810)**		**Women (n = 741)**	
	**Beta (95% CI)**	**% mediated (95% CI)***	**Beta (95% CI)**	**% mediated (95% CI)***
**Crude**				
Total effect†	0.0026 (0.0017;0.0036)		0.0020 (0.0012;0.0032)	
Direct effect†	0.0020 (0.0012;0.0030)		0.0012 (0.0006;0.0023)	
Indirect effect†	0.0005 (0.0003;0.0008)	20.5 (11.4;34.4)	0.0008 (0.0005;0.0012)	39.4 (24.4;56.9)
**Model 1**				
Total effect	0.0025 (0.0016;0.0035)		0.0018 (0.0011;0.0029)	
Direct effect	0.0017 (0.0009;0.0027)		0.0012 (0.0006;0.0021)	
Indirect effect	0.0008 (0.0005;0.0011)	30.1 (19.0;46.7)	0.0007 (0.0004;0.0010)	36.0 (21.1;54.2)
**Model 2**				
Total effect	0.0011 (0.0002;0.0021)		0.0011 (0.0006;0.0021)	
Direct effect	0.0008 (-0.0001;0.0017)		0.0008 (0.0003;0.0016)	
Indirect effect	0.0003 (0.0001;0.0005)	28.9 (11.5;107.9)	0.0003 (0.0002;0.0006)	30.2 (14.5;58.4)
**Model 3**				
Total effect			0.0011 (0.0006;0.0021)	
Direct effect			0.0007 (0.0002;0.0016)	
Indirect effect			0.0004 (0.0002;0.0007)	33.8 (17.2;61.8)

* % mediated is calculated as $\beta^{indirect} / \beta^{total} * 100$

† Total effect represents association between intrahepatic lipid (IHL) content and haemoglobin A1c (HbA1c); direct effect represents association between IHL and HbA1c not attributable to serum SHBG; indirect effect represents association between IHL and HbA1c attributable to serum SHBG (= mediation).

Model 1: adjusted for age

Model 2: additionally adjusted for BMI, alcohol intake, Dutch Healthy Diet index (DHD-13), level of education, and total physical activity

Model 3: additionally adjusted for menopausal status and use of oestrogen-containing medication

ESM Table 6. Mediation effect of serum SHBG on the association between intrahepatic lipids and (log) Matsuda-index

	**Men (n = 722)**		**Women (n = 662)**	
	**Beta (95% CI)**	**% mediated (95% CI)***	**Beta (95% CI)**	**% mediated (95% CI)***
**Crude**				
Total effect†	-0.0222 (-0.0276;-0.0179)		-0.0165 (-0.0243;-0.0119)	
Direct effect†	-0.0207 (-0.0261;-0.0164)		-0.0136 (-0.0207;-0.0095)	
Indirect effect†	-0.0015 (-0.0026;-0.0005)	6.7 (2.4;12.0)	-0.0029 (-0.0053;-0.0009)	17.8 (5.7;29.4)
**Model 1**				
Total effect	-0.0218 (-0.0268;-0.0176)		-0.0164 (-0.0242;-0.0118)	
Direct effect	-0.0193 (-0.0243;-0.0152)		-0.0136 (-0.0207;-0.0095)	
Indirect effect	-0.0025 (-0.0038;-0.0013)	11.4 (6.2;17.9)	-0.0028 (-0.0051;-0.0009)	17.0 (5.1;28.4)
**Model 2**				
Total effect	-0.0134 (-0.0182;-0.0094)		-0.0095 (-0.0150;-0.0062)	
Direct effect	-0.0127 (-0.0173;-0.0088)		-0.0087 (-0.0139;-0.0056)	
Indirect effect	-0.0007 (-0.0016;-0.0001)	5.4 (0.7;11.9)	-0.0007 (-0.0023;0.0006)	7.6 (-7.0;21.6)
**Model 3**				
Total effect			-0.0097 (-0.0150;-0.0065)	
Direct effect			-0.0081 (-0.0127;-0.0051)	
Indirect effect			-0.0016 (-0.0036;-0.0004)	16.3 (4.1;32.4)

* % mediated is calculated as $\beta^{indirect} / \beta^{total} * 100$

† Total effect represents association between intrahepatic lipid (IHL) content and Matsuda index; direct effect represents association between IHL and Matsuda index not attributable to serum SHBG; indirect effect represents association between IHL and Matsuda index attributable to serum SHBG (= mediation).

Model 1: adjusted for age

Model 2: additionally adjusted for BMI, alcohol intake, Dutch Healthy Diet index (DHD-13), level of education, and total physical activity

Model 3: additionally adjusted for menopausal status and use of oestrogen-containing medication

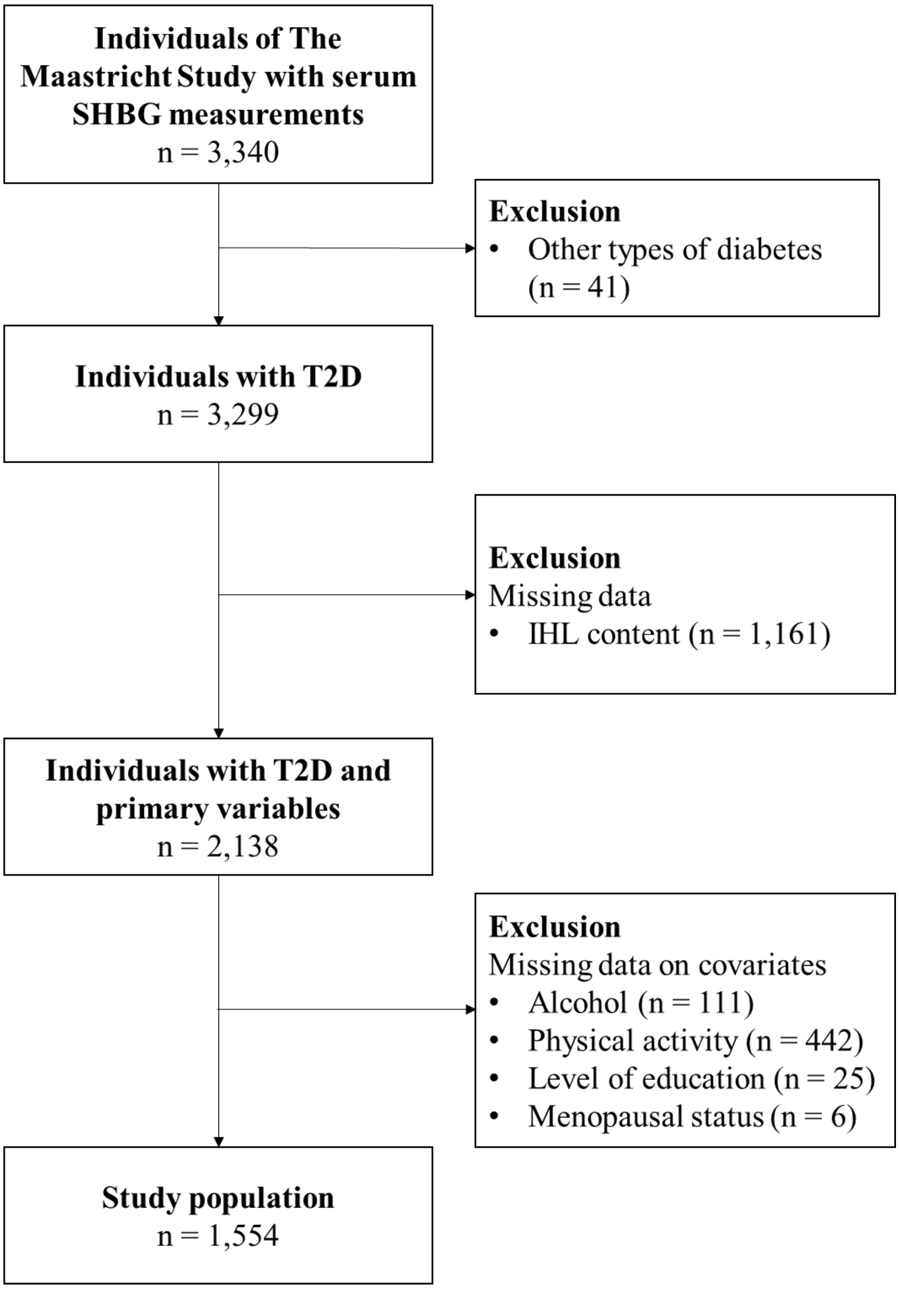

ESM Figure 1. Flowchart of study population selection

Abbreviations: IHL: intrahepatic lipid; T2D: type 2 diabetes; SHBG: sex hormone-binding globulin

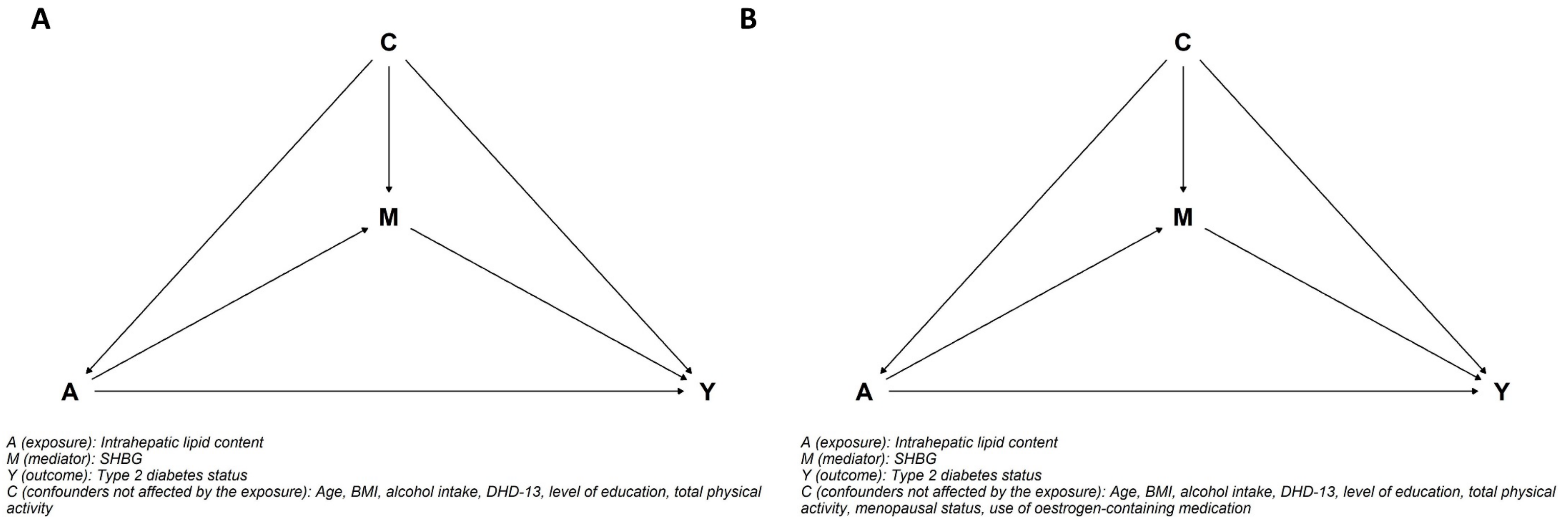

ESM Figure 2. Directed acyclic graph (DAG) illustrating the design of the mediation analyses in men (A) and women (B).
